# New York Odontological Society

**Published:** 1894-09

**Authors:** 

**Affiliations:** New York Odontological Society


### Reports of Society Meetings.

### NEW YORK ODONTOLOGICAL SOCIETY.

A REGULAR meeting of the New York Odontological Society was held on Tuesday evening, May 15, 1894, at the New York Academy of Medicine, No. 17 West Forty-third Street, New York City, with the President, Dr. Brockway, in the chair.

The minutes of the previous meeting were read by the Secretary.

### INCIDENTS OF OFFICE PRACTICE AND CASUAL COMMUNICATIONS.

*Dr. Perry.*—At one of the recent meetings, when the question of the erosion of teeth was being discussed, it was stated by some one that erosion, as we understand it, never occurs on the lingual surfaces of the teeth. I believe the gentleman was Dr. Lord.

*Dr. Lord.*—I did not mean to say that it never occurred, but that I had never seen a case.

*The President.*—Were those eroded on the labial surfaces?

*Dr. Perry.*—Not at all. They were perfectly clean and clear on the exterior surfaces.

*Dr. Jarvie.*—I do not remember ever to have seen a case of erosion with the grooves on the palatal surfaces of the teeth, but I have a set of three models, covering a period of seven and a half years, in which the incisor teeth had wasted away from erosion, and had wasted as much on the palatal and approximal surfaces as on the labial. They had wasted much more at the cutting edges than near the gums. Before erosion commenced the two centrals had had the pulps removed and gold fillings inserted in the openings made upon the palatal surfaces, and the models show just how much tooth-substance had wasted away, because the gold stood out from the teeth.

*Dr. Kimball.*—I had a curious case some years ago, which, while not in the same line, suggests itself to me at this time. A lady who had had excellent teeth came for her annual examination, and I noticed that her teeth were disappearing on the inner side. She had erosion of all her teeth, on the inner side only, as far back as the bicuspids. They looked as if they were dissolving. Questioning her pretty closely, I discovered that she was taking hydrobromic acid for headache. I asked her to bring me a sample of the acid of the diluted strength which she was in the habit of using. Covering a tooth partially with wax, I put it in the same solution, and in twenty-four hours all the exposed enamel was eaten away. The front teeth, upper and lower, where the tongue rested against them, were the only ones affected, except a little of the bicuspids; back of that there was no loss. If you ever hear of a physician prescribing hydrobromic acid without extreme precautions, you should bear this in mind.

*Dr. Walker.*—I have just shown the cast to Dr. Bonwill, and he says it is not from erosion, but from the brush.

*Dr. Bonwill.*—It is taking me away from my course to ask me about a subject like this. That which is called erosion I do not admit to be so in the way in which the term is generally used. I have seen a great deal of the injudicious use of brushes of a form which should never have been made for the use of human beings. I have yet to see a case in my practice which I can attribute to erosion, but it is generally the action of the brush upon the teeth.

People sometimes rub their teeth just as thoroughly on the inside as on the outside. Sometimes the gum and the alveolus have actually been rubbed and scrubbed away through the use of the brush. There is a great chapter to be read on this subject. I have never spoken in public about it, but I am opposed to the idea of erosion. I can show it in many cases. I have something in my pocket now that I did artificially, and you cannot tell whether it was done with a brush or acids, or what. I will tell you afterwards about it. I exhibit a brush which I guarantee will never produce this trouble.

*Dr. Jarvie.*—Does Dr. Bonwill mean that there will be no erosion or no wasting away of the teeth in mouths in which such brushes are used?

*Dr. Bonwill.*—If this brush is used, nothing of the kind will occur.

I want to say something about a point made by Dr. Howe some time ago in regard to secret recipes, why men do not give them to the profession. If he had my experience with patents, secret remedies, and nostrums, he would know that it is no use to give to the dental profession at large any invention, or to give them anything whatever, because what is everybody's business is nobody's business. Take, for instance, oxyphosphates. When the matter was first published, it was taken up by almost every manufacturer in the country. We tried one after another, and there has been an immense failure. If we could have one man upon whom we could depend, we could do something with it. I have in my pocket a piece of tin-foil which is about twenty-five years old. It was registered chemically pure tin by S. S. White himself. To-day it is simply worthless. Look at the immense number of fillings that that poor material has made. Therefore it is best for one man to take hold of a thing, and not for the whole world to go at it.

There is one other thing that I wish to speak about, and that is superficial decay. No one of you will deny to me that superficial decay can be cut out so that it will never occur again. I meet many cases, notwithstanding the close watch I keep on my patients. I have found by putting on the dam, making a partial separation, so no capillary action can take place, if I dry them thoroughly with heat and then put chloroform on those cavities, so as to take out any surplus material there, I can saturate those cavities with paraffin, and they will not decay. Take any of the old pulps that are so porous, and decay will take place again. After you get them thoroughly dry, soak them with heat and paraffin, and there is no possibility of their decaying again. If you shape your teeth properly, this will soak into the teeth and prevent further decay. Nitrate of silver is used. It is very good, of course, but it blackens the teeth. In the majority of cases, if you will let them go as long as I do with gutta-percha, you will find that your work will be successful.

When my patients go to Europe I give them a little piece of gutta-percha, and tell them if they need to go to an American dentist, they should give him that with my compliments, and tell him to put it in until they come back.

I have no trouble about the conservative treatment of the dental pulp. When men tell me they have trouble, I believe that they have exposed more pulps than they ought to.

I never put in a gold filling before I put the dam on and soak on paraffin wax until there is not a single place where it will not go in. It is impervious, and hermetically seals every part of it. There are many places that are a little too deep in superficial caries that I cannot cut out, and where I would not use paraffin wax.

*Dr. Howe.*—I would like to ask Dr. Bonwill whether he uses heat in melting the paraffin wax?

*Dr. Bonwill.*—Yes.

Dr. Bonwill then read his paper, entitled, "A New Era in Dental Practice."

(For Dr. Bonwill's paper, see page 562.)

DISCUSSION.

*Dr. Jarvie.*—I would like to know if Dr. Bonwill calls the use of the gutta-percha in this way the "new era"; that it is something entirely new, and only practised by himself, using the red base-plate gutta-percha and allowing it to remain there.

*Dr. Bonwill.*—That is a part of it.

*Dr. Jarvie.*—We are very much indebted to Dr. Bonwill for his paper in which he describes a certain system of treating decay as *new* and as original with himself, but the essayist is not the only one who has been practising that system in a certain class of cases. For young people with soft teeth I have been in the habit of doing that very same thing, cutting out the approximal decay and filling in solidly with red gutta-percha, and allowing it to remain there in many cases for a year, and sometimes two years or more, according to circumstances. Upon the removal of what filling may be left, I find the structure of the tooth under the gutta-percha very much harder, and in a condition to receive a metallic filling, generally gold. I have done this for years in cases of young people and poorly calcified teeth, and I think you will find that there are many gentlemen in the room who have done that same thing. I do not consider it new or novel.

*Dr. Lord.*—The hour is now late, and as there is much in this paper that some of us will not agree to, particularly the use of amalgam to the extent that it is recommended, we do not want to have it go out to the world without being properly discussed and the questionable features pointed out. We should take care of the bad things, and the good things will take care of themselves. This is an old adage, but one that generally holds true.

I think it would be wise to postpone the discussion of this paper until another time.

On motion of Dr. Howe it was decided to postpone discussion until the paper had appeared in print.

*Dr. Bonwill.*—I would not have you understand that I was the first man who ever used gutta-percha, or the first one to use oxy-phosphate. That would be ridiculous. I call your attention to the use of these things in a way in which you have not used them. No man has separated the teeth as I have done. The best evidence of it is in the separators that you use, and the way you do it. There is one thing that I do tell you that you have not noticed, and that is in regard to the placing of the first permanent molar. The farther back you keep it, the less regularity you have. It is a grand point. With all of your intelligence, you have not studied that in connection with articulation.

*Dr. Bogue.*—I take it we all have different kinds of practice coming to us. The teeth have been so treated that Dr. Bonwill sets himself to work to find a remedy for the treatment. He uses gutta-percha as being the best separator that he can find, and, having a great many of these cases, he waits for three or four months, or even a year, until he has space enough; and then he has a position of teeth, upper and lower, in their relation to each other that permits him to contour, and to contour in such a way that the teeth are no longer painful and no longer accumulate food between them.

On motion of Dr. Lord, a vote of thanks was extended to Dr. Bonwill for his kindness in presenting the paper.

Adjourned.

John I. Hart, D.D.S.,
*Editor New York Odontological Society.*

